# The regulatory mechanisms of cerium oxide nanoparticles in oxidative stress and emerging applications in refractory wound care

**DOI:** 10.3389/fphar.2024.1439960

**Published:** 2024-08-02

**Authors:** Lijun Yi, Lijian Yu, Shouying Chen, Delong Huang, Cheng Yang, Hairui Deng, Yiheng Hu, Hui Wang, Zhongjian Wen, Yiren Wang, Yu Tu

**Affiliations:** ^1^ Department of General Surgery, Luzhou People’s Hospital, Luzhou, China; ^2^ School of Nursing, Southwest Medical University, Luzhou, China; ^3^ Wound Healing Basic Research and Clinical Application Key Laboratory of Luzhou, Luzhou, China; ^4^ Department of Clinical Medicine, Southwest Medical University, Luzhou, China; ^5^ School of Basic Medical Science, Southwest Medical University, Luzhou, China; ^6^ Department of Medical Imaging, Southwest Medical University, Luzhou, China; ^7^ People’s Hospital of Nanjiang, Bazhong, China; ^8^ Department of Oncology, The Affiliated Hospital of Southwest Medical University, Luzhou, China

**Keywords:** cerium oxide nanoparticles, oxidative stress, refractory wound, wound care, biocompatibility, toxicity

## Abstract

Cerium oxide nanoparticles (CeNPs) have emerged as a potent therapeutic agent in the realm of wound healing, attributing their efficacy predominantly to their exceptional antioxidant properties. Mimicking the activity of endogenous antioxidant enzymes, CeNPs alleviate oxidative stress and curtail the generation of inflammatory mediators, thus expediting the wound healing process. Their application spans various disease models, showcasing therapeutic potential in treating inflammatory responses and infections, particularly in oxidative stress-induced chronic wounds such as diabetic ulcers, radiation-induced skin injuries, and psoriasis. Despite the promising advancements in laboratory studies, the clinical translation of CeNPs is challenged by several factors, including biocompatibility, toxicity, effective drug delivery, and the development of multifunctional compounds. Addressing these challenges necessitates advancements in CeNP synthesis and functionalization, novel nano delivery systems, and comprehensive bio effectiveness and safety evaluations. This paper reviews the progress of CeNPs in wound healing, highlighting their mechanisms, applications, challenges, and future perspectives in clinical therapeutics.

## Graphical Abstract

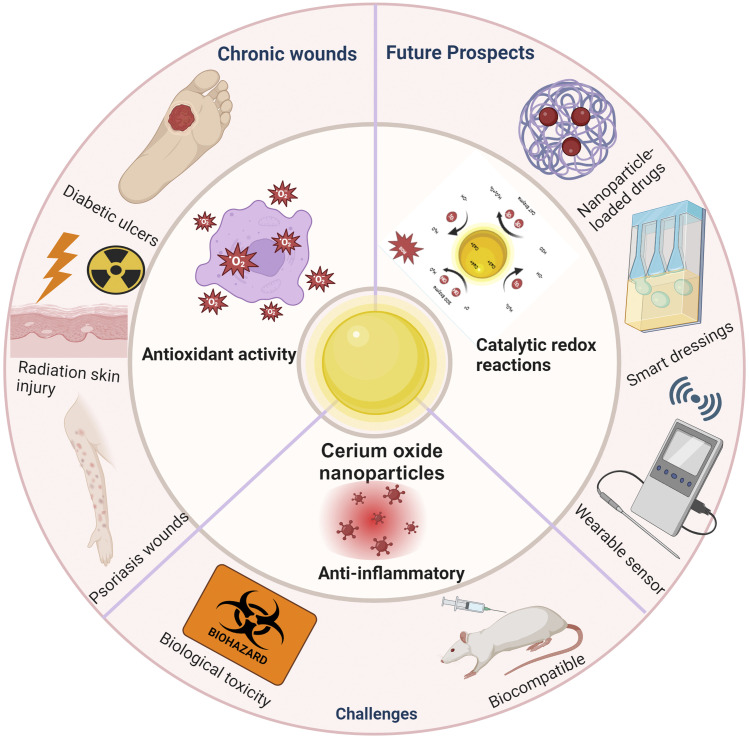

## 1 Introduction

Wound healing is a complex biological process that involves four stages: inflammation, granulation tissue formation, wound contraction, and remodeling (Wang et al., 2018). These stages are continuous and overlapping. When the wound repair process is disrupted, it can lead to delayed or difficult healing, often referred to as chronic wounds if the repair time exceeds 1 month (Bates-Jensen, 1999). The formation of chronic, non-healing wounds is closely related to the local microenvironment of the wound (such as temperature, humidity, pH value, oxygen content, and biofilm formation), with oxidative stress playing a significant role (Clinton and Carter, 2015). Oxidative stress refers to the imbalance between free radicals and antioxidants produced during the wound healing process, which has been proven to delay the healing process (Deng et al., 2021). Therefore, regulating oxidative stress in wounds is crucial for promoting rapid and effective healing. In recent years, with the wide application of nanotechnology in the medical field, cerium oxide nanoparticles (CeNPs) have become a focus in wound treatment due to their unique antioxidant properties (Dhall and Self, 2018). CeNPs demonstrate strong antioxidant capacity by mimicking the function of various enzymes in the body, such as superoxide dismutase and catalase (Saifi et al., 2021; Corsi et al., 2023; Pandey et al., 2024). They regulate the dynamic balance between Ce (III) and Ce (IV) oxidation states, effectively removing excess free radicals and thus protecting cells from oxidative damage (Forouzanfar et al., 2023; Chen et al., 2024). This study first reviews the chemical composition and structural characteristics of CeNPs, followed by an exploration of their antioxidant stress mechanisms at the cellular biology level, including their impact on cell signaling. Subsequently, the discussion shifts to the practical applications of CeNPs in treating oxidative stress-induced non-healing wounds. Finally, the current research progress is summarized, and future research directions are envisioned to provide new therapeutic strategies for the treatment of chronic wounds.

## 2 Basic characteristics of cerium oxide nanoparticles

### 2.1 Chemical composition and structure

Cerium oxide nanoparticles (CeNPs) are nanomaterials composed of the rare earth metal cerium (CeO_2_) in the form of oxides (Humaira et al., 2023). In the nanoparticles, cerium is present in the III and IV oxidation states, conferring excellent antioxidant and catalytic properties to CeNPs. In oxygen-rich environments, cerium ions are predominantly Ce (IV), forming stable CeO_2_ structures. Under oxygen-poor or reducing environment, some Ce (IV) ions are transformed into Ce (III) ions, accompanied by the release of oxygen. The high oxygen storage capacity of cerium oxide mainly originates from its redox cycle between Ce (III)/Ce (IV). Through its reversible oxygen ion storage/release capacity, cerium oxide can absorb and release oxygen, thus regulating the rate and equilibrium of redox reactions. When Ce (III) ions in cerium oxide are reduced to Ce (IV) ions, oxygen ions are released from the lattice. This oxygen-releasing ability makes cerium oxide effective in promoting redox reactions in industrial catalytic processes. It has been shown that the size of cerium oxide particles also affects their oxygen storage and release capacity. As the size of CeNPs decreases, the concentration of oxygen vacancies on the surface or inside the particles increases, with a corresponding increase in Ce (III) content. This leads to an increase in the lattice constant of cerium oxide particles, which in turn affects their oxygen storage and release capacity. On the nanoscale, the proportion of Ce (III) increases with decreasing particle size, which is crucial for its catalytic effect (Shin et al., 2022). CeNPs usually exhibit a cubic fluorite crystal structure, in which each cerium atom is surrounded by eight oxygen anions, and each oxygen atom is located at a tetrahedral site (Lakshmi et al., 2021). The cubic fluorite crystal structure gives cerium oxide excellent catalytic properties and thermal stability due to its stability and high ordering. The dissociated oxygen and vacancies can act as active sites to adsorb other substances, thus affecting the catalytic performance of cerium oxide, which is the key to the catalytic ability of CeNPs, allowing them to switch between CeO_2_ and CeO_2-x_ through the loss of oxygen atoms and/or electrons. Moreover, in cerium oxide, the presence of lattice defects improves its resistance to compression and hardness, making it widely used in fields such as solid oxide fuel cells and optical materials. The physicochemical properties of these nanoparticles, such as size, shape, surface properties and charge, can be precisely controlled by various synthesis methods for specific biomedical applications (Humaira et al., 2023).

### 2.2 Physical properties

Cerium oxide nanoparticles are typically between 1 and 100 nm in size, which gives them a high surface area to volume ratio, which enhances their surface reactivity and directly affects their efficiency as catalysts (Baldim et al., 2020). Optically, the refractive index of cerium oxide varies with wavelength and generally ranges from 1.9 to 2.3. Cerium oxide has a distinct absorption peak in the near-infrared band, which is most typical at about 1,400 nm. There is also a small amount of absorption in the ultraviolet and visible ranges, but compared to its absorption intensity in the near-infrared range, it is small and does not vary significantly. Cerium oxide nanoparticles have exceptional optical properties, absorbing, emitting and scattering light. This has led to a wide range of applications in the fields of optical materials and photocatalysis, such as the preparation of optical sensors, photocatalysts, and optical coatings, making it potentially useful in biomedical imaging (Kolmanovich et al., 2023). From the point of view of electronic properties, cerium oxide nanoparticles have good electrical conductivity and high thermal stability. Its conductivity mechanism is related to lattice defects, surface defects, and doping. The smaller the grain size of cerium oxide nanoparticles, the more lattice defects and surface defects, the better the electrical conductivity; at the same time, increasing the doping concentration within a certain range can also significantly improve the electrical conductivity of cerium oxide. Its unique electronic structure also endows CeNPs with magnetic properties, making them useful in medical imaging techniques such as magnetic resonance imaging (MRI) (Eriksson et al., 2018). In addition, CeNPs have a large specific surface area, which increases the contact area with other substances. This gives CeNPs higher reactivity and catalytic properties. CeNPs can rapidly transition between different oxidation states, a property that makes them useful in applications such as catalysts, oxidizing agents, and reducing agents. CeNPs have good antioxidant properties, and can be used as an antioxidant to prevent the oxidation and corrosion of materials. The shape and size of CeNPs affects their bioactivity and drug delivery efficiency. Nanoparticles of specific shapes may penetrate cell membranes more easily or release drugs more efficiently inside cells (Yadav, 2022). For example, smaller CeNPs are more easily phagocytosed by cells, which may increase their efficacy in targeted therapy (Wu et al., 2018). In conclusion, cerium oxide nanoparticles excel in optical, electronic, and other physical properties, and these properties make it promising for a wide range of applications in several fields, such as catalysts, solar cells, gas sensors, antioxidant materials, and photocatalysis.

### 2.3 Surface characteristics and modification

The surface characteristics of cerium oxide nanoparticles are crucial for their behavior in biological systems. The surfaces of CeNPs can undergo various modifications to enhance their biocompatibility and therapeutic effects (Wang et al., 2022). Such modifications often involve adding hydrophilic or hydrophobic layers, targeting ligands, or altering their surface charge. Hydrophilic modifications, like coating with polyethylene glycol (PEG) or polysaccharides, can reduce protein adsorption, thereby diminishing the immunogenicity of CeNPs and extending their circulation time in the bloodstream (Brandão Da Silva Assis et al., 2024). Hydrophobic modifications can increase the permeability of CeNPs in cell membranes (Casals et al., 2017). Surface modifications can endow CeNPs with specific cellular targeting capabilities, enabling them to reach damaged tissues more effectively. It has been found that bis-[3-(triethoxysilyl) propyl] tetrasulfide silane (TESPT) is one of the most widely used silanes in the rubber industry (Vilmin et al., 2014). It is a bifunctional silane with six ethoxyl groups that bind to the surface of the nanoparticles. TESPT molecules also have a tetrasulfide group involved in the vulcanization of the rubber (Neethirajan et al., 2022). This will form a chemical bond between the nanoparticles and the rubber chains, thus improving the properties of the nanocomposites. Based on previous studies, TESPT was found to be a suitable silane for surface modification of nanoparticles to improve the dispersion of nanoparticles in rubber polymers and to impart properties to the rubber matrix. The presence of a limited number of hydroxyl groups on the surface of CeNPs offers the possibility of chemical bonding with silane molecules. The surface modification of CeNPs by TESPT was investigated in a study by Biuk Afshari et al. (2022). The surface modification of CeNPs by TESPT was investigated using sol-gel method. The nanoparticles were vaporized to increase their functionality and to improve their chances of reacting with TESPT molecules. The results were as follows: due to the low functionality of CeNPs, a higher silane concentration (X = 10) was required to increase the silane grafting rate. A pH of 10 in water/ethanol solution is the optimum hydrolysis condition for surface modification of CeNPs by TESPT (Nanda, 2016; Biuk Afshari et al., 2022). Additionally, altering the surface charge can impact the interactions between CeNPs and cell membranes, affecting their phagocytosis and endocytosis.

### 2.4 Biomimetic activity

Cerium oxide nanoparticles have attracted widespread attention in the biomedical field due to their unique enzyme-mimicking activities (Deng et al., 2021). These nanoparticles can mimic the functions of natural antioxidant enzymes like superoxide dismutase (SOD) and catalase, effectively eliminating excess reactive oxygen species (ROS). The biomimetic activity of CeNPs is primarily attributed to the dynamic balance between surface Ce (III) and Ce (IV) oxidation states and their ability to rapidly transition between these states under varying redox conditions (Baldim et al., 2020). In mimicking biological enzymes, CeNPs also exhibit potential antioxidant actions against various detrimental intracellular ROS, positioning them as materials with regenerative therapeutic potential, particularly in wound healing and anti-inflammatory treatments (Xu et al., 2023). Moreover, these properties also offer CeNPs vast application prospects in drug delivery, biosensing, and tissue engineering (Sadidi et al., 2020; Pramanik et al., 2022; Park et al., 2023).

### 2.5 Nanoparticle dynamics and stability

The biokinetic properties of cerium oxide nanoparticles determine their distribution, metabolism, and excretion within organisms. The size, surface characteristics, and modifications of CeNPs influence their transport and localization in the body, thereby determining their effectiveness in targeted therapy (Shin et al., 2022). The stability of CeNPs refers to their ability to maintain functional and structural integrity, which is crucial for their applications as long-term antioxidants or drug carriers (Hasanzadeh et al., 2019). In the biological environment, CeNPs may be affected by various biochemical processes, including protein binding, phagocytosis, and dissolution. These processes can alter the surface properties and catalytic activities of CeNPs, thereby impacting their biocompatibility and therapeutic effectiveness. Investigating the stability of CeNPs in simulated biological settings and real biological systems is essential for predicting and optimizing their performance in clinical applications. Advanced analytical techniques, such as near-edge X-ray absorption fine structure spectroscopy (NEXAFS) and dynamic light scattering (DLS), are employed to thoroughly assess the stability of CeNPs (Verma et al., 2023).

### 2.6 Methods for synthesizing nanoparticles and controlling particle size

Cerium oxide nanoparticles are synthesized by a variety of methods (Dhall and Self, 2018). Traditional synthesis methods include Precipitation, which synthesizes Spherical nanoparticles with a particle size of 15 nm (Pelletier et al., 2010); Hydrothermal, which synthesizes Octahedral nanoparticles with a particle size of 5 nm (Rojas et al., 2012); Solvothermal, which synthesizes ∼8 nm Polyhedral nanoparticles (Domingues et al., 2023); Spray Pyrolysis, which synthesizes Cubic nanoparticles with a particle size of ∼17 nm (Demokritou et al., 2013); while these methods can help determine shape and size, many of the traditional methods are less biocompatible. Currently, the commonly used synthesis methods are green synthesis methods, including Plant-mediated, which synthesizes Spherical nanoparticles with a particle size of 36 nm (Charbgoo et al., 2017); Fungus-mediated, which synthesizes Spherical nanoparticles with a particle size of 5 nm (Lu et al., 2022); Polymer-mediated, which synthesizes ∼2 nm Spherical nanoparticles (Fifere et al., 2024); Nutrient-mediated, which synthesizes Spherical nanoparticles with a particle size of 25 nm (Ahmed et al., 2021). These CeNPs are more stable with higher water dispersibility and high fluorescence properties.

## 3 Antioxidant stress response mechanism of cerium oxide nanoparticles

**FIGURE 1:**
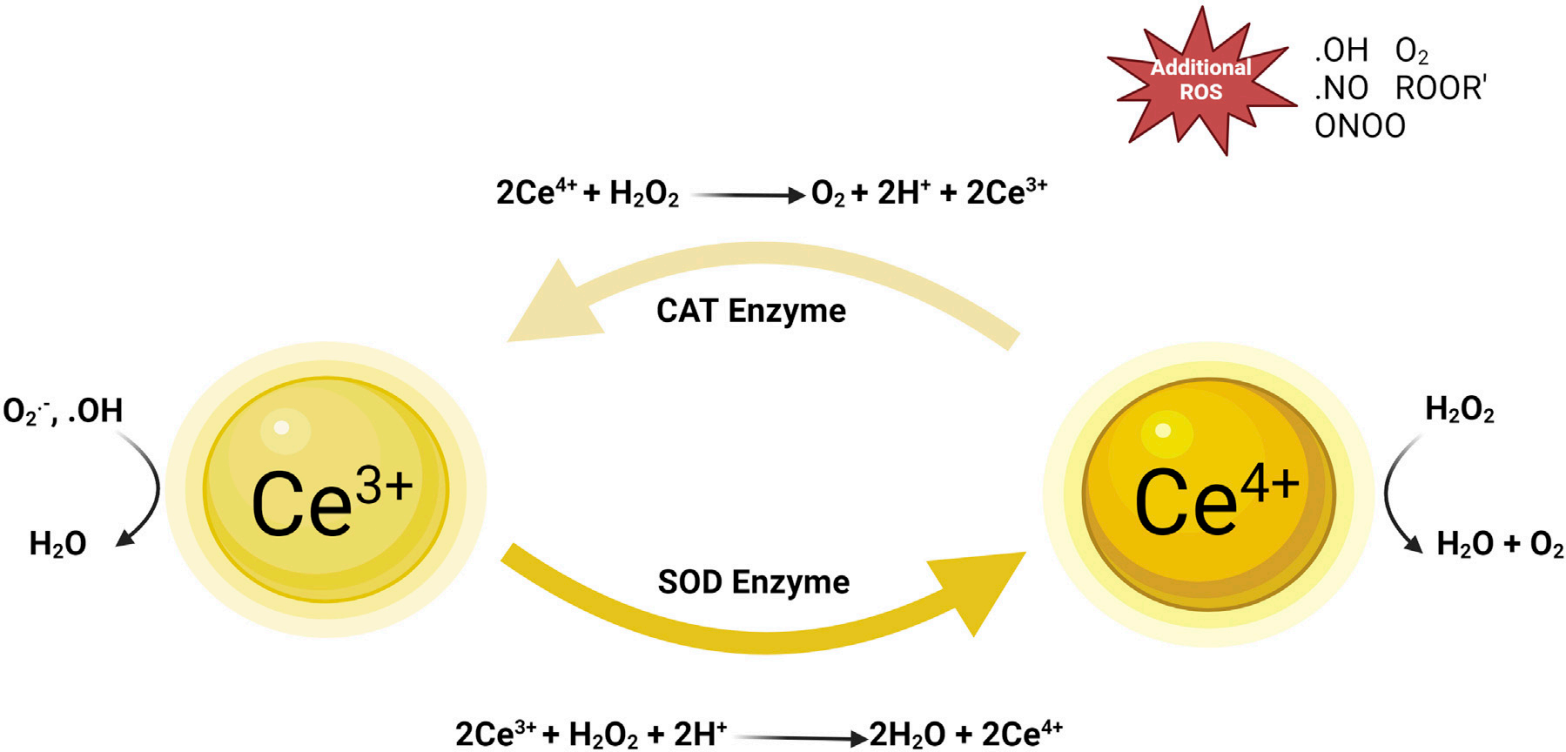
Mechanism diagram of nano-cerium oxide particles against oxidative stress. Reproduced with permission (Zeng et al., 2018).

Reactive oxygen species (ROS) are essential byproducts of cellular metabolism, including superoxide anion (O^2–^), hydrogen peroxide (H_2_O_2_), and hydroxyl radical (-OH) (Sies, 2015). ROS play a crucial role in regulating cellular signaling pathways, which typically control cell proliferation, growth, cycle, and death. Moreover, ROS are significant in antimicrobial activity (Lord et al., 2021). During the phagocytosis of pathogens, high levels of ROS such as O^2−^ and H_2_O_2_ are produced within phagosomes, leading to the generation of hypochlorous acid (HOCl), whose potent oxidizing capability can kill internalized pathogens (Dunnill et al., 2017). However, when ROS levels exceed cellular antioxidant defenses, either through an increase in ROS or a decrease in cellular antioxidant capacity, an imbalance in oxidative activity occurs. The resulting oxidative stress causes oxidative damage to cellular macromolecules such as lipids, proteins, and DNA, closely associated with the development and exacerbation of chronic wounds like diabetic foot ulcers, radiodermatitis, and psoriasis (Dal Magro et al., 2021). External administration of exogenous antioxidants or inhibition of local oxidative stress responses may restore pathological states. Cerium oxide nanoparticles, due to their structural characteristics, can assist in clearing various toxic reactive oxygen species produced at wound sites, thereby promoting the healing of chronic wounds. CeNPs mainly exhibit antioxidant stress responses through the following mechanisms (Dhall and Self, 2018) (Figure 1).

### 3.1 Direct scavenging of reactive oxygen free radicals and catalyzing redox reactions

A key role of cerium oxide nanoparticles (CeNPs) in biomedicine is their ability to directly scavenge reactive oxygen free radicals through their surface redox activity. The unique nanoscale effects of CeNPs are not only due to their size but also due to the reversible conversion between cerium atoms in +3 and +4 oxidation states on their surface. This redox dynamic characteristic enables CeNPs to mimic the function of superoxide dismutase (SOD), catalyzing the conversion of superoxide anion (O^2−^) into oxygen (O_2_) and hydrogen peroxide (H_2_O_2_), the latter further being reduced to water (H_2_O), thus reducing oxidative damage to cells (Zengin et al., 2022). In the catalytic redox reaction, Ce (III) on the surface of cerium oxide nanoparticles can capture oxygen atoms from H_2_O_2_ and convert it to water (H_2_O), while Ce (III) is oxidized to Ce (IV). Under reducing conditions, Ce (IV) can be reduced back to Ce (III) by scavenging of H_2_O_2_ radicals, or the intracellular redox of cerium oxide nanoparticles can be maintained by selecting suitable reducing agents such as metallic reducing agents (e.g., iron, zinc, etc.), nonmetallic reducing agents (e.g., sulfur, iodine, etc.), ionic reducing agents (e.g., ferrous ions Fe^2+^), and certain organic compounds (e.g., alcohols, aldehydes, etc.) in the redox cycle. This electron transfer reaction enhances the ability of CeNPs to eliminate ROS, effectively protecting cells from oxidative stress (Baldim et al., 2020). Additionally, possible defect sites on CeNPs’ surface, like oxygen vacancies, provide extra sites for redox activity, serving as platforms for capturing and transferring electrons, further enhancing their antioxidant effects (Goujon et al., 2021). Thus, the physicochemical properties of CeNPs play a crucial role in cellular protection and wound healing processes, especially in controlling oxidative stress and promoting tissue repair. Understanding this mechanism can aid in developing new therapeutic strategies that leverage CeNPs’ antioxidant properties for treating wounds and inflammation.

### 3.2 Enhancing cellular antioxidant defense capabilities and anti-inflammatory effects

CeNPs have a unique mechanism and role in antioxidant and anti-inflammatory (Hayat et al., 2014). CeNPs can promote the dissociation and nuclear translocation of the Nrf2 transcription factor, a key regulator in the cellular defense mechanism that activates the expression of various antioxidant response genes, enhancing the cell’s own defense mechanisms (Carvajal et al., 2019; Xiong et al., 2023). Activation of the Nrf2 pathway by CeNPs increases the expression of endogenous antioxidant enzymes like superoxide dismutase (SOD), catalase (CAT), and glutathione peroxidase (GPx), which are directly involved in the scavenging of ROS. CeNPs also enhance the biosynthesis of the antioxidant molecule glutathione (GSH), the primary non-enzymatic antioxidant within cells, crucial for neutralizing ROS and protecting cells from oxidative damage (Corsi et al., 2023). Furthermore, CeNPs may regulate various signaling pathways related to ROS clearance, including modulation of intracellular calcium signaling and the MAPK signaling pathway, which are closely associated with the cellular antioxidant response (Pesaraklou and Matin, 2020).

Additionally, by affecting the synthesis and secretion of cytokines, CeNPs can mitigate local and systemic inflammatory responses caused by the excessive accumulation of inflammatory factors (Azari et al., 2019). CeNPs can reduce the activity of signaling molecules in key inflammatory pathways, such as inhibiting the activation of nuclear factor κB (NF-κB), a transcription factor that regulates the expression of various inflammatory factors, whose inhibition can significantly reduce the production of inflammatory factors like TNF-α, IL-1β, and IL-6 (Kobyliak et al., 2017). CeNPs may intervene in various cell signaling pathways related to inflammation, including but not limited to the MAPK pathway, JAK/STAT pathway, and PI3K/Akt pathway, all of which play central roles in inflammatory responses (Kim et al., 2021). Furthermore, due to their antioxidant properties, CeNPs can reduce the accumulation of ROS within cells, thereby indirectly lowering oxidative stress-induced inflammatory signaling (Wu and Ta, 2021).

## 4 Application of cerium oxide nanoparticles in oxidative stress-related refractory wounds

### 4.1 Diabetic ulcers

**FIGURE 2:**
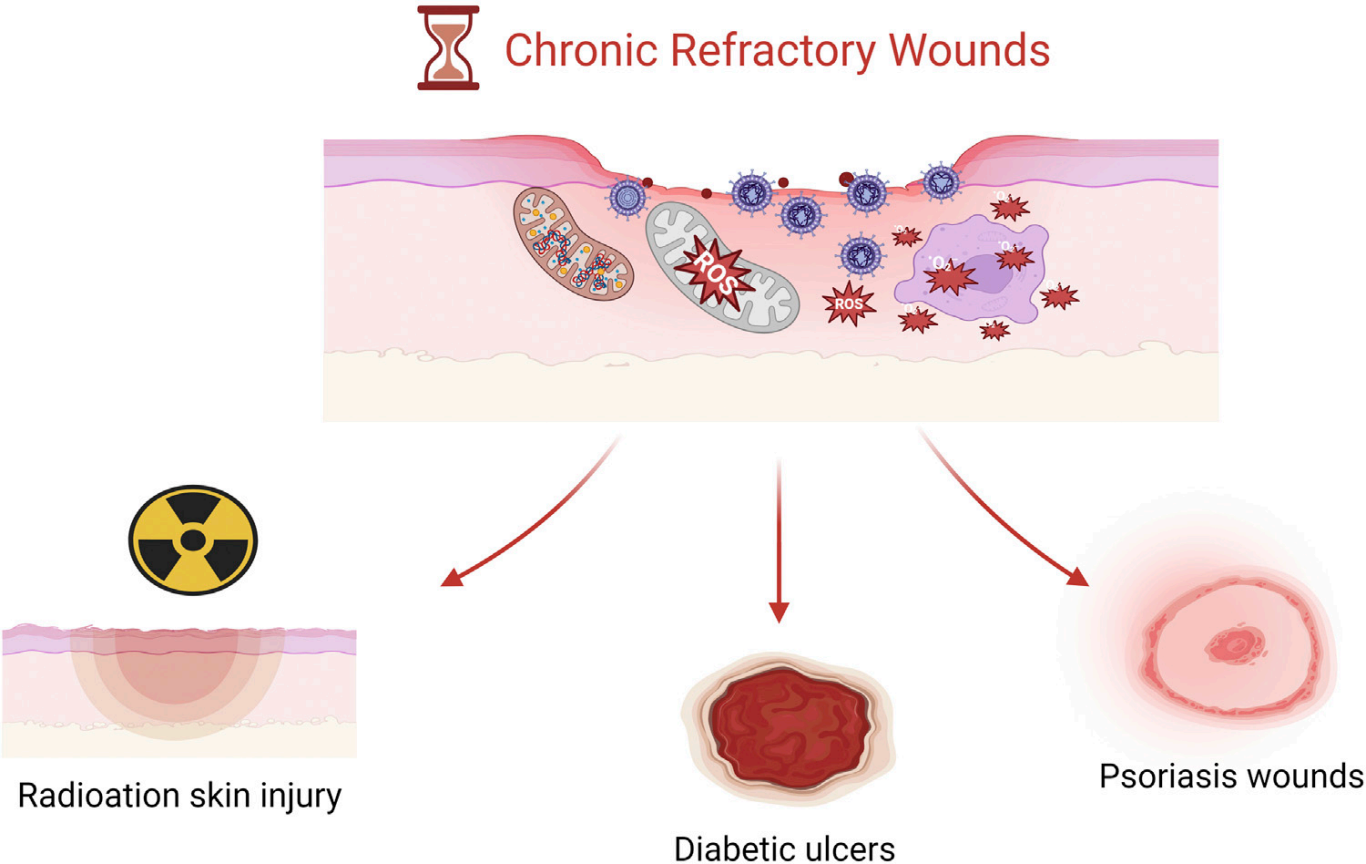
Three types of chronic refractory wound images.

Diabetic ulcers are a major complication in patients with diabetes. Increased oxidative stress under hyperglycemic conditions leads to tissue damage and an impediment in cellular repair functions (Liu et al., 2022). Additionally, diabetic-induced neuropathy directly impacts the neural innervation and microcirculation required during the wound healing process, exacerbating the chronic inflammation state of ulcers (Chang and Nguyen, 2021). Zgheib et al. (2019) conducted an animal study on diabetic mouse skin ulcers using cerium oxide nanoparticles tagged with microRNA-146a, showing that treatment with microRNA-146a cerium oxide significantly improved wound healing rates. Furthermore, Sener et al. (2020) developed a CNP-miR146a amphiphilic cryogel, which not only possesses injectability and self-healing properties but also allows for sustained release of CNP-miR146a over time, thereby reducing wound inflammation, promoting wound healing, and shortening healing time in diabetic mice. Kobyliak et al. (2019) conducted a case report study applying cerium oxide nanoparticles to diabetic foot ulcers with neuropathy, demonstrating CNP’s excellent antibacterial activity, anti-inflammatory properties, and ability to penetrate deep into wound tissues, reducing oxidative damage and thereby protecting regenerative tissue, indicating its potential for local treatment of diabetic foot ulcers (Figure 2).

### 4.2 Radiation-induced skin injury

Radiation-induced skin injury is a primary complication of tumor radiotherapy or occupational exposure, posing a significant challenge in oncological care. The main cause of radiation-induced skin injury is the destruction of double-stranded DNA structures by high-energy radiation, leading to a series of cellular and tissue damages, creating issues like wound hypoxia, oxidative stress responses, and downregulation of DNA repair proteins (Wang et al., 2023). Zhou et al. (2022) investigated a novel type of mesoporous silica (MS) firmly anchoring and dispersing cerium oxide nanoparticles, forming an MS-CeO_2_ nanocomposite for treating a radiation-induced skin injury mouse model. The study found that this composite exhibited excellent activity in inhibiting radiation-induced ROS and HIF-1α activation. Beyond direct applications in treating radiation-induced skin injuries, Verma and colleagues developed advanced flexible and moldable X-ray shielding bandages using *in situ* synthesized polygonal cerium oxide nanoparticles/MWCNT nanocomposites, offering high shielding performance and flexibility, potentially reducing radiation exposure during CT scans or occupational protection, thus minimizing damage to normal tissues (Verma et al., 2023) (Supplementary Material S1).

### 4.3 Psoriasis wounds

Psoriasis is a chronic inflammatory skin disease closely related to oxidative stress, where antioxidation and inhibition of keratinocyte abnormal proliferation are key strategies for treatment (Boehncke and Schön, 2015). Wu et al. (2020) used cyclodextrin-modified CeO_2_ nanoparticles as multifunctional nanozymes in a mouse model of psoriasis, finding that CeO_2_ nanoparticles exhibited excellent superoxide dismutase and catalase-mimicking activities, preventing ROS-mediated damage, and also demonstrating promising drug delivery efficacy. Wang et al. (2019) conducted a study using a nano-delivery system composed of all-trans retinoic acid (TRA), triphenylphosphine (TPP)-modified cerium oxide nanoparticles, flexible nano-liposomes, and gel applied in mouse animal models and cellular experiments. The results showed that the synthesized composite material had excellent mitochondrial targeting capability and valence state conversion ability related to the scavenging of reactive oxygen species (ROS). Results from EGF-induced and H_2_O_2_-induced models *in vitro* indicated effective reduction in inflammation levels and alleviation of oxidative stress in HaCat cells.

Although the production of a significant amount of reactive oxygen species (ROS) in the wound microenvironment is beneficial for wound healing, unregulated excess production of ROS can cause damage to DNA, proteins, lipids, and even cells (Dunnill et al., 2017; Wang et al., 2019). Furthermore, increased ROS production in damaged tissues can trigger harmful effects, such as cellular aging, fibrotic scarring, and uncontrolled inflammation, leading to the development of chronic wounds (Wang et al., 2024). Therefore, optimal balance of ROS in the wound microenvironment plays a crucial role in promoting wound healing. Cerium oxide nanoparticles can directly scavenge reactive oxygen free radicals and catalyze redox reactions, as well as enhance cellular antioxidant defense capabilities and anti-inflammatory effects, demonstrating their potential as a therapeutic strategy for regenerative wound healing (Supplementary Material S2).

## 5 Scaffolds and composites containing CeNPs

### 5.1 GelMA-CeNPs

A biodegradable gelatin methacryloyl (GelMA) hydrogel patch containing cerium oxide nanoparticles (CeNPs) was developed to promote diabetic wound healing. The morphology, physico-mechanical properties, free radical scavenging activity, *in vitro* cell proliferation and *in vivo* diabetic wound healing activity of the patches were thoroughly characterized. The GelMA-CeNPs, with high porosity, excellent exudate uptake, and outstanding free radical scavenging activity, enhanced the proliferation of 3T3 fibroblasts and HaCaT keratinocytes, promoted re-epithelialization, and accelerated wound healing. The overall results suggest that CONP-loaded GelMA hydrogels are very promising materials for the development of clinically relevant patches for the treatment of diabetic wounds (Augustine et al., 2021).

### 5.2 PHBV-CeNPs

PHBV-CeNPs had high porosity, enhanced tensile strength due to the presence of CeNPs, enhanced cell viability and adhesion of HOECs and HMECs, promoted keratinocyte migration in the scratch assay, vascularization in chicken embryo angiogenesis assay, enhanced cellular infiltration and granulation tissue formation, and facilitated diabetic wound healing. The study suggests that CeO_2_-doped PHBV membranes have a strong potential to be used as wound dressings to enhance cell proliferation and vascularization and to promote diabetic wound healing (Augustine et al., 2020).

### 5.3 CBMA or SBMA/HEMA/miRNA146a-conjugated CeNPs

CBMA or SBMA/HEMA/miRNA146a-conjugated CeNPs are injectable, self-healing, non-cytotoxic, and sustainably release miRNA146a-conjugated CeNPs to accelerate the wound healing process, enhance the biomechanical properties of the skin, increase the expression of miR146a and type 1 collagen genes, and downregulate the pro-inflammatory cytokines IL-6 and CXCL2. A biomaterial system of amphoteric ionized cryogels (gels formed at freezing temperatures) containing CNP-miR146a that are topically applied, injectable, self-repairing, and provide sustained release of therapeutic molecules. These cryogels consist of CBMA or SBMA and HEMA and do not contain chemical cross-linking agents. The properties of the gels can be manipulated by changing the type and ratio of monomers. These materials have demonstrated efficacy and feasibility *in vivo* in a diabetic mouse wound healing model. Overall, these materials have high potential for application in wound therapy due to their ease of production, anti-fouling properties, durability, topical application and slow release mechanism (Sener et al., 2020) (Supplementary Material S3).

## 6 Current application challenges and prospects

### 6.1 Biocompatibility and toxicity

Before clinical application, cerium oxide nanoparticles (CeNPs), as potential therapeutics, must undergo rigorous biocompatibility and toxicity assessments. While they have demonstrated excellent antioxidant and anti-inflammatory effects *in vitro*, their long-term stability, metabolic pathways, and potential toxicity due to accumulation *in vivo* are not fully understood (Kalyanaraman et al., 2019; Goujon et al., 2021). Biocompatibility issues involve the distribution, absorption, metabolism, and excretion of CeNPs in the body, which collectively determine their safety during therapeutic processes (Sarnatskaya et al., 2020). *In vivo*, CeNPs may affect normal cellular functions, including cell proliferation, migration, and apoptosis, and inappropriate size, dosage, and surface modifications may exacerbate these effects (Casals et al., 2020). Current studies indicate that under certain conditions, CeNPs may induce oxidative stress and inflammatory responses, particularly at higher doses or with prolonged exposure (Adebayo et al., 2020). Moreover, some forms of CeNPs might exhibit cytotoxicity by generating ROS, related to their surface properties and charge (Forest et al., 2017).

Toxicity of nanoparticles is currently a major issue. In biomedical applications, the assessment of nanoparticle toxicity is a critical factor that must be thoroughly addressed. CeNPs have been widely used in biomedical applications, including skin tissue engineering and wound healing studies. Researchers are working to reduce their potential toxicity while maintaining their biological activity by chemically modifying and encapsulating cerium oxide nanoparticles. Despite promising results in various studies, a number of studies have revealed the toxicity of these nanoparticles. Aalapati et al. demonstrated the accumulation of CeNPs in lung tissues of CD1 mice as well as the pulmonary toxicity of these nanoparticles after nasal inhalation exposure (Aalapati et al., 2014). Attia et al. (2022) demonstrated that hepatic accumulation of CeNPs at standard therapeutic doses did not have significant cytotoxicity in healthy animal toxicity. At high doses (hundreds of mg/kg BW), CeNPs showed toxicity in rodents, although hepatoprotective effects were induced at doses up to 1 mg/kg BW. Similarly, CeNPs used at higher doses *in vitro* have been reported to impair cell viability. In addition to the dose, the biokinetics of CeNPs will largely depend on the physicochemical characteristics and exposure routes, including concentration, nanoparticle size, exposure time, preparation method, exposure route, and cell/tissue type. Cheng et al. (2021) evaluated the viability of HaCaT cells cultured in GelMA/polybutylene-based hydrogels loaded with different concentrations of CeNP (1, 10, 100, 1,000 μg/mL, and 10 mg/mL) using CCK-8 assay. The results showed that hydrogels containing 100 μg/mL CeNPs had no significant effect on cell viability. However, hydrogels containing 1,000 μg/mL and 10 mg/mL significantly reduced the viability of HaCaT cells, demonstrating the potentially cytotoxic high concentration of admixed CeNPs. Wu et al. (2019) demonstrated the toxicity of CeNPs after repeated intranasal drops in mice by two different sizes of CeNPs, and found that the CeNPs permeated through the air-blood barrier after lung loading. The nanofilaments were further transferred to secondary target organs, mainly the liver and spleen. Systemic accumulation of CeNPs eventually triggered lipid peroxidation in multiple organs. In general, smaller nanofilaments caused more severe lung injury compared to larger nanofilaments, but the systemic toxicity was similar. Therefore, addressing the toxicity and safety of cerium oxide nanoparticles is a critical and topical issue for biomedical development.

### 6.2 Effective drug loading and delivery

The effective drug loading and delivery capabilities of cerium oxide nanoparticles (CeNPs) in drug delivery systems are crucial for their clinical application. CeNPs have the potential to function as drug carriers, capable of binding, encapsulating, or adsorbing various drug molecules, including small molecules, peptides, proteins, and genetic materials (Hosseini and Mozafari, 2020). The challenge lies in ensuring that these drugs are stably loaded onto CeNPs and effectively released at the target site. Researchers are exploring different surface modification techniques, such as polymer coating and targeting ligand attachment, to enhance the stability and delivery efficiency of CeNPs as drug carriers (Popov et al., 2018; Fu et al., 2022). These strategies aim to enhance the affinity between CeNPs and drugs, improve their distribution within organisms, and provide targeted capabilities for specific cells or tissues. Moreover, controlled drug release from CeNPs is another critical aspect. Ideally, drugs should be released in response to specific physiological conditions, such as changes in pH, enzyme activity, or redox states, to maximize therapeutic effects while minimizing systemic side effects. Current research on CeNPs in delivery systems focuses on improving their penetration capabilities to enable effective drug passage through biological barriers like skin or the blood-brain barrier (Bignon et al., 2017; Dal Magro et al., 2021). Researchers are also striving to understand and optimize the biokinetics of CeNPs to ensure accurate drug delivery to lesion sites (Hasanzadeh et al., 2019; Fayyazi et al., 2023). Facing these challenges, future research needs to focus on developing more advanced nanoparticle synthesis methods, more intelligent drug loading strategies, and more efficient targeted delivery technologies. Through interdisciplinary cooperation and the integration of knowledge in nanoscience, medicinal chemistry, and bioengineering, it is expected to overcome these obstacles and promote the application of CeNPs in the treatment of oxidative stress-related reactions (Rzigalinski et al., 2017; Wang et al., 2022).

### 6.3 Development of multifunctional compounds

In the field of nanomaterial research, developing compounds with multiple functions is a current trend (Liu et al., 2023). These multifunctional compounds not only provide basic antioxidant and anti-inflammatory effects but can also combine other therapies such as chemotherapy, targeted therapy, or phototherapy to enhance treatment effects and offer personalized approaches for complex disease states (Lord et al., 2021). The development of multifunctional CeNPs involves surface modification or doping to introduce additional therapeutic functionalities (Kalyanaraman et al., 2019). For example, doping with magnetic materials can endow CeNPs with capabilities as contrast agents in magnetic resonance imaging (MRI) or play roles in magnetotherapy (Kolmanovich et al., 2023; McDonagh et al., 2023). Additionally, CeNPs can be combined with drug molecules, small interfering RNA (siRNA), or other gene-editing tools to bring new strategies for treating cancer or genetic diseases (Kolanthai et al., 2022). Another important aspect of multifunctional compounds is their ability to respond to specific physiological signals, like changes in pH or the presence of enzymes, to achieve controlled drug release, minimizing impacts on normal tissues (Liu et al., 2023). Future directions include strategies combining the photothermal properties of CeNPs with photodynamic therapy and developing nanocomposites capable of precisely delivering multiple drugs, playing an increasingly important role in future healthcare. Gao et al., (2023) exploited the electrostatic interactions between CeO_2_ nanoparticles, cerium 5-hydroxytryptophan oxide and hyaluronic acid to develop an active MPO-targeted hyaluronic acid/cerium hydroxytryptamine oxide nanoenzymes (HA-5-HT@CeO_2_). Based on the dual targeting effect of MPO and macrophage CD44^+^ receptor to localize the site of colonic inflammation via electrostatic interaction, CeO2 nanoparticles, together with several similar enzymes, were used for the elimination of reactive oxygen species such as O_2_, H_2_O_2_, and ˙OH, and targeted repair of the intestinal epithelial barrier through the elimination of inflammatory factors. In in vitro pharmacodynamics and *in vivo* DSS-induced acute colitis animal model studies, HA-5-HT@CeO_2_ further reduced inflammation and treated ulcerative colitis compared with conventional drugs. In addition, active targeting of MPO inflammation can deliver the drug to the exact site of pathogenesis and minimize the side effects of the drug. HA-5-HT@CeO_2_ is a novel and promising drug for the treatment of ulcerative colitis (Gao et al., 2023).

### 6.4 Smart wound dressings and wearable sensors

In chronic wound care, developing smart dressings and wearable sensors that integrate drug delivery systems and wireless communication technologies for real-time monitoring of the wound microenvironment, early diagnosis, and on-demand treatment represents a major future trend (Sun et al., 2021). Nanomaterials, with their nanoscale size, grain boundary structures, and surface effects, can provide superior performance compared to ordinary dressings and have the advantage of gradually replacing traditional dressings (Wang et al., 2019). Due to their excellent antioxidant properties and structural characteristics, cerium oxide nanoparticles (CeO_2_NPs) can play a key role in wound care, with their surfaces easily binding to other biomolecules (Ma et al., 2019; Allu et al., 2023). For example, He et al. (2022) developed PFD/CeO_2_ NC capsule dressings with complex structures using a layer-by-layer method, which demonstrated good biocompatibility and ROS scavenging ability in cell experiments, and could be directly applied to wounds. Animal experiments further proved that this dressing accelerated epithelialization, reduced ROS and TGF-β levels, improved the arrangement and proportion of collagen fibers, thereby promoting wound repair and anti-scarring. Additionally, Wang et al. (2021) created intelligent antibacterial wound dressings by loading amino phenylboronic acid (ABA) modified gold nanoclusters (A-GNCs) onto bacterial cellulose films. These dressings, when used to treat wounds infected with multidrug-resistant bacteria, could display the remaining amount of A-GNCs nanodrug *in situ* through colorimetry, with A-GNCs emitting bright orange fluorescence under UV excitation. Hence, the BC-A-GNCs nanocomposite material would show a gradual decrease in orange fluorescence intensity as A-GNCs are released, providing an appropriate timing for dressing change. Such “smart” nanoparticles can respond to changes in the microenvironment, such as ROS, pH, specific enzymes, bacterial toxins, etc. Since cerium oxide nanoparticles are sensitive and responsive to ROS, future strategies could further integrate cerium oxide nanoparticles into wound dressings to enable wound monitoring for better wound healing.

## 7 Conclusion

Cerium oxide nanoparticles (CeNPs) have shown significant therapeutic efficacy in wound treatment, primarily due to their robust antioxidant capabilities. By mimicking the activity of natural antioxidant enzymes, CeNPs can mitigate oxidative stress and reduce the production of inflammatory mediators, thereby accelerating wound healing. Moreover, CeNPs have demonstrated potential in treating inflammation and infections across various disease models, particularly excelling in managing oxidative stress-induced chronic wounds such as diabetic ulcers, radiation-induced skin injuries, and psoriasis wounds. Despite positive progress in laboratory research, CeNPs still face several challenges in clinical applications, including but not limited to biocompatibility, toxicity, effective drug delivery, and the development of multifunctional compounds. Strategies to address these challenges, such as improving the synthesis and functionalization of CeNPs, developing novel nanodelivery systems, and conducting more comprehensive bioeffectiveness and safety assessments, will be focal points for future research.
