# Supplementary material for: The regulatory mechanisms of cerium oxide nanoparticles in oxidative stress and emerging applications in refractory wound care
Source: Front Pharmacol. 2024 Aug 2;15:1439960. doi: 10.3389/fphar.2024.1439960 (PMC11327095; doi:10.3389/fphar.2024.1439960)
Supplement: Supplementary file 1 [file DataSheet1.pdf]

Supplementary Figure 1:

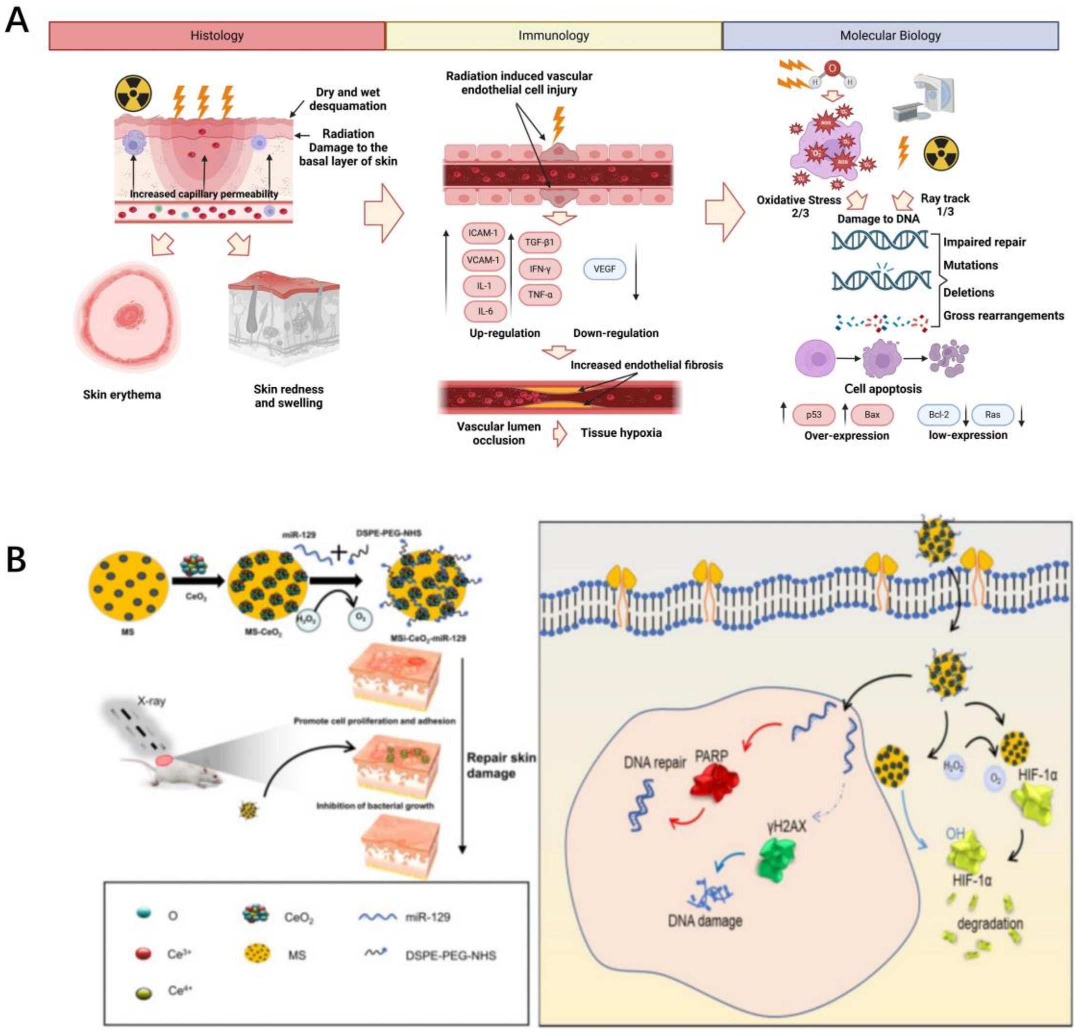

Schematic diagram of potential mechanisms of acute radiation skin injury. Reproduced with permission [58]. B. Multifunctional mesoporous silica-cerium oxide nanozymes facilitate miR129 delivery for high-quality healing of radiation-induced skin injury. Reproduced with permission [59].

Supplementary Figure 2

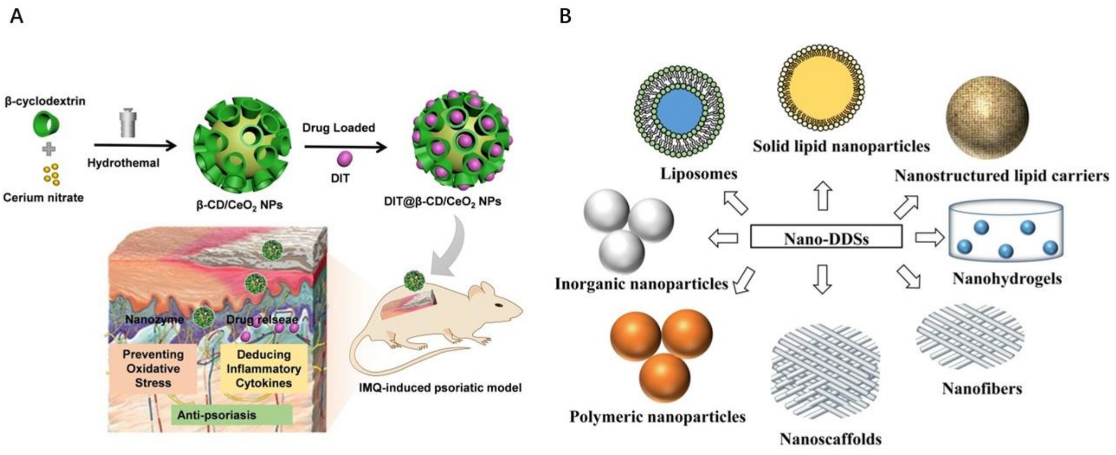

A. Schematic interpretation of the design of β-cyclodextrin capped ceria nanoparticles as a nanozyme loaded with dithranol for the combinational therapy of psoriasis. Reproduced with permission [61]. B. Nano-drug delivery systems in skin regeneration and wound treatment. Reproduced with permission [62].

Supplementary Figure 3

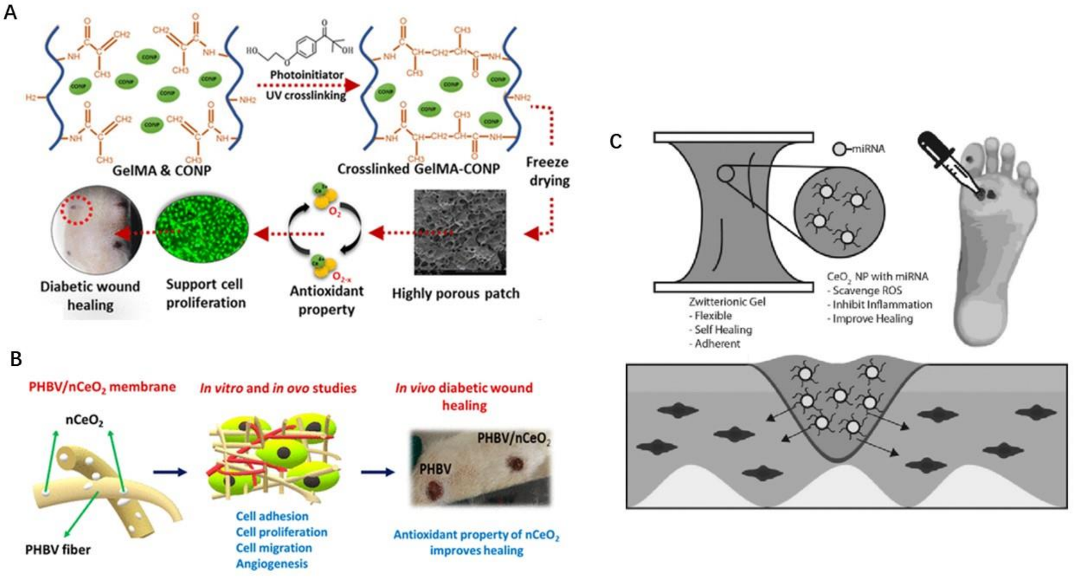

A. Biodegradable gelatin methacryloyl (GelMA) hydrogel patch combined with cerium oxide nanoparticles (CONP) promotes diabetic wound healing. Reproduced with permission [64]. B. Development of a novel NCEO 2-containing electrospun poly (3-hydroxybutyrate co-3-hydroxyvalerate) (PHBV) membrane for diabetic wound healing applications. Reproduced with permission [65]. C. Biomaterial system for amphiphilic ionic ice gels (gels formed below freezing) of CNP-miR146a. Reproduced with permission [56].
